# Targeting *PLA2G7* ameliorates high-fat diet–induced pulmonary injury in obese mice, uncovering a key mechanistic link to obesity-associated COPD

**DOI:** 10.1186/s12931-026-03540-6

**Published:** 2026-02-06

**Authors:** Zhi-Heng Li, Mei-Yu Lv, Xin Zhang, Bao-cai Wang, Yao Wang, Li-Xia Qiang, Xiangshun Li, Wenchao Shi, Xin-yu Guo, Xi-Qiao Sang

**Affiliations:** 1 Department of Respiratory Medicine, The Fourth Affiliated Hospital of Harbin Medical University, Harbin, 150081 China; 2 Department of General Surgery, Shanghai Sixth People’s Hospital Affiliated to Shanghai Jiaotong University, Shanghai, 200233 China; 3 Department of Medical Oncology, Harbin Medical University Cancer Hospital, Harbin, 150001 China; 4 Department of Thoracic Surgery, The Fourth Affiliated Hospital of Harbin Medical University, Harbin, 150081 China; 5 Department of General Surgery, The Fourth Affiliated Hospital of Harbin Medical University, Harbin, 150001 China; 6 Key Laboratory of Preservation of Human Genetic Resources and Disease Control in China (Harbin Medical University), Ministry of Education, Harbin, 150081 China

**Keywords:** Chronic obstructive pulmonary disease, Obesity, *PLA2G7*, Alveolar Macrophages, Arachidonic acid, *NLRP3*

## Abstract

**Background:**

Obesity is a major risk factor for chronic obstructive pulmonary disease (COPD); however, the precise molecular pathways remain poorly defined, and it is uncertain whether severe obesity by itself can trigger COPD-like pathology. PLA2G7 has been identified as a pathogenic gene in COPD, yet the molecular mechanisms by which PLA2G7 contributes to disease development remain to be elucidated.

**Methods:**

To investigate the role of PLA2G7 in obesity-related chronic obstructive pulmonary disease (COPD), we employed clinical specimens as well as in vivo and in vitro models, integrating multi-omics approaches with genetic and pharmacological interventions. Key methodologies included protein and gene expression analyses (Western blotting,, immunohistochemistry, ELISA assay, qRT-PCR), assessment of oxidative stress and lipid peroxidation (ROS and BODIPY staining), histological evaluation (H&E Staining, Oil Red O Staining, AB-PAS staining), transmission electron microscopy, micro-computed tomography (micro-CT), and pulmonary function tests in mice. Furthermore, molecular docking and molecular dynamics simulations were performed to explore potential molecular interactions of PLA2G7.

**Results:**

Using a diet-induced obesity model, this study revealed that obesity alone elicits COPD-like pulmonary changes, defined by weight-dependent alveolar injury, elevated airway mucus secretion, and reduced lung function. Transcriptomic analysis of lung tissues from obesity-associated COPD patients and obese mice identified macrophage-derived phospholipase A2 group VII (*PLA2G7*) as a key regulator of disease pathogenesis. Genetic deletion or pharmacological suppression of *PLA2G7* was found to reduce obesity-associated COPD-like pathology. Mechanistically, high-fat stimulation induced upregulation of *PLA2G7* in macrophages, leading to increased release of arachidonic acid (AA). The higher AA levels promoted the accumulation of lipid-derived reactive oxygen species (ROS) and stabilized *NLRP3* mRNA by reducing its degradation. This process activated the inflammasome and triggered pyroptosis, thus driving pulmonary inflammation and contributing to COPD development.

**Conclusions:**

*PLA2G7* acts as a key mediator of obesity-associated COPD and represents a promising therapeutic target for preventing obesity-related lung injury.

## Highlights

1. Obese mice exhibit body weight–dependent COPD-like pulmonary alterations.

2. *PLA2G7* acts as a key regulator in obesity-driven COPD

3. Upregulation of *PLA2G7* enhances the generation of arachidonic acid

4. Arachidonic acid slows *NLRP3* mRNA degradation

5. *NLRP3* activation promotes pyroptosis, mediating obesity-related COPD pathogenesis.

## Introduction

Chronic obstructive pulmonary disease (COPD) is expected to remain the third leading cause of mortality worldwide by 2025 and is the fifth largest contributor to the global economic burden of disease [1–3]. Although cigarette smoking remains the most significant risk factor, nearly half of COPD cases are due to non-smoking causes, with obesity recognized as an important factor [4, 5]. Epidemiological data show that the prevalence of overweight and obesity is significantly higher in COPD patients compared to underweight individuals, with approximately 19.1% of hospitalized COPD patients classified as obese [6–8]. Moreover, the incidence of obesity in COPD populations continues to increase [9]. This obesity-associated COPD phenotype is characterized by nutritional excess, metabolic disturbances such as increased body mass index (BMI), chronic inflammation, and potentially genetic factors [10, 11]. However, the exact molecular mechanisms through which obesity, especially severe obesity, causes COPD to develop and progress are still not fully understood [12].

Excessive lipid deposition, a characteristic feature of obesity, is recognized as a major trigger for oxidative stress and persistent inflammation, both key aspects of COPD pathology [13, 14]. Pulmonary macrophages play a crucial role in regulating airway inflammation and serve as key metabolic sensors, improving inflammatory responses in lipid-rich environments [15, 16]. However, the specific enzyme–lipid–ROS signaling pathways that promote macrophage-mediated lung damage in COPD are not yet fully understood [17].

Phospholipase A2 group VII (*PLA2G7*), also termed lipoprotein-associated phospholipase A2, is a secreted enzyme predominantly expressed by macrophages [18–20]. Located at the intersection of lipid metabolism and inflammatory regulation, *PLA2G7* hydrolyzes oxidized phospholipids to release free fatty acids, a bioactive lipid that affects cellular lipid flow and immune responses [21]. Besides its role in lung disease, *PLA2G7* has been associated with various pathological processes, including immune regulation through *PD-L1*-mediated suppression of CD8⁺ T cells, fibrotic signaling via the lysophosphatidic acid (*LPA*)/*LPA₂* pathway, triggering metabolic inflammation by releasing lipid mediators, and affecting susceptibility to regulated cell death, such as Ferroptosis [22–24]. Despite its broad functional importance and potential for therapy, the role of *PLA2G7* in macrophage dysfunction under obesity-related lipotoxic stress and its involvement in COPD progression remain mostly unexplored [25].

To investigate this unresolved issue, lung tissues from obese COPD patients were analyzed in combination with a diet-induced obesity mouse model and macrophage-focused in vitro systems. Through genetic and pharmacological manipulation, *PLA2G7* was identified as a key mediator of obesity-related COPD. Obese mice showed weight-dependent alveolar damage, mucus hypersecretion, impaired pulmonary function, increased *PLA2G7* expression, and accumulation of AA. Mechanistic insights revealed that lipid overload enhanced *PLA2G7* expression in macrophages, subsequently raising AA levels. This cascade increased susceptibility of membrane lipids to peroxidation, intensified lipid ROS generation, activated the NLRP3 inflammasome, and triggered pyroptotic cell death, thus worsening pulmonary inflammation and contributing to COPD progression.

These results reveal a previously uncharacterized enzyme–lipid–ROS signaling axis that maintains the lipotoxic pulmonary microenvironment typical of obesity-associated COPD. *PLA2G7* and its downstream mediators are emphasized as vital pathogenic drivers, offering potential opportunities for therapeutic intervention in this metabolically driven COPD phenotype.

## Materials and methods

### Human samples

#### Study population and tissue collection

This cohort study was conducted at the Fourth Affiliated Hospital of Harbin Medical University, following approval from the institutional ethics committee (Approval No. 2025-IRB-58). Written informed consent was obtained from all participants before sample collection. Lung tissue specimens were collected from two sources:

(i) histologically normal parenchyma (> 5 cm from the tumor margin) in patients undergoing lung cancer resection, and.
(ii) bulla samples obtained during therapeutic lung volume reduction surgery.

Individuals with a history of cigarette smoking, interstitial pulmonary fibrosis, or autoimmune disorders were excluded from participation.

#### Diagnostic criteria

COPD: Diagnosis was based on the GOLD 2025 guidelines, defined as post-bronchodilator FEV₁/FVC < 0.70.

Hyperlipidemia: Determined according to ESC/EAS 2023 recommendations, characterized by LDL-C ≥ 3.4 mmol/L or triglycerides ≥ 1.7 mmol/L confirmed on two separate occasions.

### Animal models

Male C57BL/6 mice (6–8 weeks old) were purchased from Huachuang Xinnuo Pharmaceutical Technology Co., Ltd. (Jiangsu, China) and housed under specific pathogen-free (SPF) conditions with a 12 h light/dark cycle and unrestricted access to food and water. Animals were randomly divided into experimental groups and provided either a normal chow diet (NC; D12450J, 10% kcal from fat) or a high-fat diet (HFD; D12492, > 60% kcal from fat).

For in vivo suppression of *PLA2G7*, mice received tail vein injections of recombinant adenoviral vectors expressing *PLA2G7*-targeted shRNA. After 3–4 weeks, knockdown efficiency was confirmed by Western blotting, showing > 70% reduction in protein expression compared to the controls. The *PLA2G7* shRNA adenoviral vector was designed and obtained from Applied Biomaterials Co., Ltd. (Jiangsu, China).

All animal procedures were reviewed and approved by the Institutional Animal Care and Use Committee of the Fourth Affiliated Hospital of Harbin Medical University (Approval No. 2025-DWSYLLCZ-66) and conducted in accordance with institutional animal welfare standards.

### Cell culture and lipid overload model

The murine alveolar macrophage cell line MH-S (Cell Bank of the Chinese Academy of Sciences, Shanghai, China) was cultured in RPMI1640 (KGL1501, KeyGEN BioTECH, China) supplemented with 10% heat-inactivated fetal bovine serum (12,352,200, Sigma-Aldrich, USA) at 37 °C in a humidified incubator with 5% CO₂. To induce lipid overload, cells were treated with different concentrations of oleic acid (OA) (KC006, KunChuang Biotechnology, China) ranging from 0 to 1.2 mmol/L for 24 h. Cell viability was measured using the CCK-8 assay (C0037, Beyotime, China), and a dose–response curve was generated. The half-maximal inhibitory concentration (IC₅₀) was calculated, and 0.75 mmol/L OA for 24 h was selected for further experiments.

### Cell transfection

Transient transfection was performed using Lipofectamine™ 3000 reagent (L3000015, Invitrogen, USA) following the provided protocol. After 6 h of incubation, the culture medium was replaced with fresh complete medium. The efficiency of gene silencing or overexpression was evaluated by Western blot analysis 24 to 48 h after transfection. Small interfering RNAs (*siRNAs*) and *NLRP3* expression plasmids were designed and supplied by Applied Biological Materials Inc. (Jiangsu, China). The sequences used were as follows:

*siPLA2G7*, sense 5′-CGUUGGUUGUACAGACUUAAUTT-3′.

### Transcriptome sequencing and metabolomics

High-throughput transcriptome profiling and untargeted metabolomic analysis using LC–MS/MS were performed to detect differentially expressed genes (DEGs) and metabolite profiles. All transcriptomic and metabolomic tests were conducted at Applied Protein Technology (Yiwu, China).

### Immunofluorescence

Paraffin-embedded tissue sections were processed for immunofluorescence staining using standard protocols. Primary antibodies included rabbit anti-PLA2G7 (1:100, ER1914-69, Huabio, China), rabbit anti-CD68 (1:1000, ab303565, Abcam, UK), and rabbit anti-NLRP3 (1:100, ET1610-93, Huabio, China). Images were obtained using the Pannoramic MIDI II imaging platform (3DHISTECH, Hungary).

### Immunohistochemistry (IHC)

Mouse lung tissues were fixed in 4% paraformaldehyde (PFA), embedded in paraffin, and sectioned for IHC analysis. Primary antibodies used were rabbit anti-GSDMD-N (1:1000, HA721144, HUABIO, China), rabbit anti-CASPASE-1 (1:1000, ET1608-69, HUABIO, China), and rabbit anti-ASC (1:500, 83,858–3-RR, Sangon Biotech, China). Images were captured using a fluorescence microscope (CX23, Olympus, Japan).

### ROS assay

Fresh lung tissues were cryosectioned into 10-µm slices, and cells were cultured on confocal dishes. Samples were incubated with dihydroethidium (DHE) working solution (S0064S, Beyotime Biotechnology, China) at 37 °C for 20 min (dark). Tissue sections were imaged using a fluorescence microscope (CX23, Olympus, Japan), and cell fluorescence was visualized with a confocal laser scanning microscope (Nikon, Japan). Fluorescence intensity was quantified using ImageJ software.

### BODIPY staining

Cells were stained with BODIPY working solution (C2053S, Beyotime, China) according to the manufacturer's instructions. Green fluorescence was detected using a confocal laser scanning microscope with excitation/emission settings of 493 nm and 503 nm, respectively. Fluorescence intensity was quantified using ImageJ software.

### Superoxide dismutase (SOD) and malondialdehyde (MDA) detection

Lung tissues and cells were homogenized in PBS (tenfold volume) using ultrasonic disruption, while serum samples were analyzed directly. SOD activity was measured using the Total SOD Activity Assay Kit (A001-3, Nanjing Jiancheng Bioengineering Institute, China). MDA levels were determined using the MDA Assay Kit (S0131S, Beyotime Biotechnology, China) according to the respective protocols.

### Histological staining and triglyceride measurement

Paraffin-embedded lung tissue sections were stained with hematoxylin and eosin (H&E) using a commercial H&E Staining Kit (C0105S, Beyotime Biotechnology, China) according to the manufacturer's instructions. Frozen lung tissue sections were stained with Oil Red O using an Oil Red O Staining Kit (C0158S, Beyotime Biotechnology, China), and images were captured under a light microscope. Triglyceride (TG) levels in mouse lungs and serum were quantified with a TG Assay Kit (A110-1–1, Nanjing Jiancheng Bioengineering Institute, China).

### AB-PAS Staining

AB-PAS Staining: Paraffin-embedded lung sections were first deparaffinized, rehydrated, and stained with Alcian Blue (pH 2.5) for 30 min, followed by periodic acid–Schiff (PAS) staining for 15 min. These sections were counterstained with hematoxylin, dehydrated, and mounted for analysis. Goblet cell proliferation and mucus production were examined using light microscopy.

### Transmission electron microscopy (TEM)

Lung tissues (~ 1 mm^3^) from each group were fixed overnight in glutaraldehyde, then fixed in 1% osmium tetroxide. The samples were dehydrated through a graded ethanol series, infiltrated, embedded in epoxy resin, and processed for imaging. Ultrathin sections were prepared, stained as per standard protocols, and images were acquired using a transmission electron microscope.

### Micro-CT scan

High-resolution micro-computed tomography (micro-CT) imaging of mouse lungs and whole bodies was performed using a PerkinElmer Quantum GXII scanner. The X-ray tube voltage was set to 50 kVp, and images were acquired at a spatial resolution of 50 µm. Reconstruction was performed using Hiscan Reconstruct software, while quantitative analyses were performed using Hiscan Analyzer software.

### Pulmonary function test

Sodium pentobarbital was administered to induce anesthesia in all mice, followed by endotracheal intubation. Respiratory function was measured using the Buxco DSI platform (Buxco DSI, St. Paul, MN, USA) and analyzed through indicators such as forced vital capacity (FVC), peak expiratory flow (PEF), forced expiratory volume at 20 ms (FEV20), and functional residual capacity (FRC).

### ELISA assay

The concentrations of PLA2G7, NLRP3, GSDMD-N, CASPASE-1, ASC, IL-1β, and IL-18 in lung tissues, serum, or cells were measured using ELISA kits (Jianglai Biological, China) according to the manufacturer's instructions.

### Western blotting

Protein expression was evaluated through immunoblotting using established protocols. Primary antibodies derived from rabbits included: PLA2G7 (ER1914-69, Huabio, China), NLRP3 (15,101, Cell Signaling Technology, USA), GSDMD-N (HA721144, Huabio, China), CASPASE-1 (ET1608-69, Huabio, China), ASC/TMS1 (83,858–3-RR, Proteintech, China) and GAPDH (AB0036, Abways Technology, China). The secondary antibody used was goat anti-rabbit IgG (1:10,000, AS014, ABclonal Technology, China).

### RT-PCR

Total RNA was isolated from cultured cells using TRIzol reagent (Invitrogen). Complementary DNA (cDNA) was synthesized using a reverse transcription kit (Takara) as per the manufacturer's instructions. RT-PCR was performed using specific primers targeting the genes of interest, with actin serving as the housekeeping reference gene. The primer sequences were as follows:

- *NLRP3* forward: 5′-CCGTCTACGTCTTCTTCCTTTC-3′,
- reverse: 5′-CGCAGATCACACTCCTCAAATA-3′;
- *actin* forward: 5′-ATTGGCAACGAGCGGTTCC-3′,
- reverse: 5′-AGCACTGTGTTGGCATAGAGG-3′.

### Molecular docking

Protein structures of ZFP36, DCP2, and Regnase1 were retrieved from the UniProt database. Ligand molecules were acquired from PubChem using their CAS identifiers and converted from SDF to MOL2 format using Open Babel. Docking simulations were performed with AutoDock 4.2 and AutoDock Tools 1.5.7, and the conformations with the lowest binding energies were selected for visualization and analysis.

### Molecular dynamics simulation of the AA–NLRP3 mRNA complex

Initial docking analyses were performed using the HDOCK server, and the resulting AA–*NLRP3* mRNA assemblies were prepared for molecular dynamics (MD) simulations. The complexes were solvated in a TIP3P water box and neutralized with 0.15 M NaCl. MD simulations were performed using GROMACS v2022.04 with the CHARMM36 force field. After energy minimization, equilibration was conducted for 500 ps under NVT and NPT ensembles at 300 K and 325 K, respectively, followed by extended production runs of 100 ns. The trajectories were analyzed for root mean square deviation (RMSD), root mean square fluctuation (RMSF), radius of gyration (Rg), and intramolecular hydrogen bonds (HBOND-in). Structural renderings and graphical analyses were performed using PyMOL, VMD, and GraphPad Prism.

### Statistical analysis

Data were analyzed using GraphPad Prism v9.0 (GraphPad Software, USA). Two groups were compared using unpaired Student's *t*-tests. One-way analysis of variance (ANOVA) was used for single-factor analyses, while two-way ANOVA with Tukey's post hoc test was applied for comparisons involving two independent variables. Statistical significance was set at *p* < 0.05. Results are presented as mean ± standard error of the mean (SEM). Levels of significance were denoted as *p* < 0.05, *p* < 0.01, *p* < 0.001, and *p* < 0.0001.

## Results

### PLA2G7 upregulation in obese patients with COPD

**Fig. 1:**
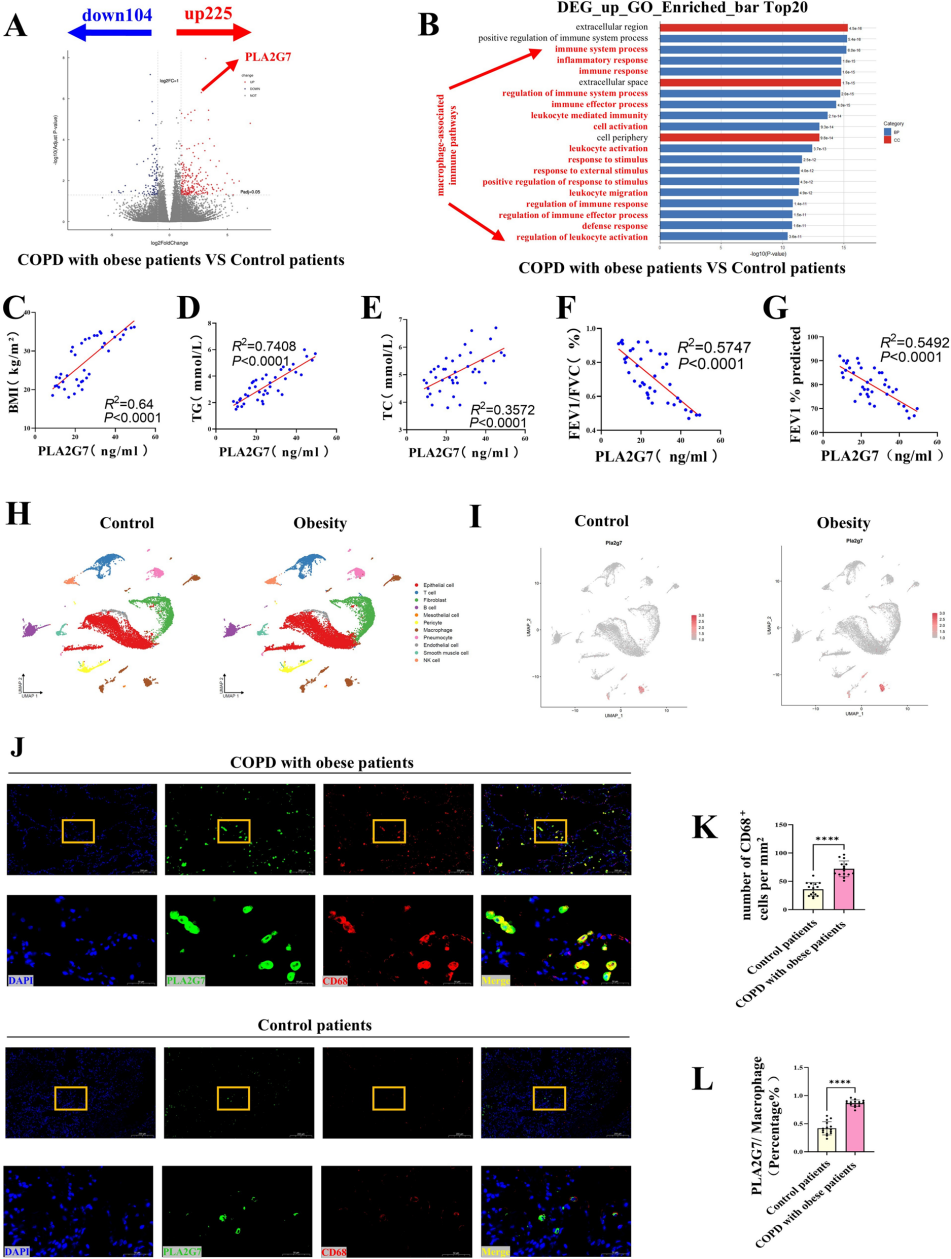
*PLA2G7* upregulation in obese patients with COPD. **A** Volcano plot showing 225 differentially expressed genes (DEGs) significantly upregulated in lung tissues from obese COPD patients. **B** Functional enrichment analysis of upregulated DEGs highlights immune-related pathways, particularly macrophage activation. **C**–**G** Correlation analyses reveal that PLA2G7 expression positively correlates with BMI and serum lipids (TC and TG) and negatively correlates with lung function indices (FEV1/FVC and FEV1% predicted). **H**-**I** Single-cell clustering and cell type identification in high-fat diet (HFD) and control groups. **J**–**L** Immunofluorescence staining localizes PLA2G7 expression to pulmonary macrophages in obese COPD lungs

Transcriptomic analysis of lung tissues from obese COPD patients revealed 225 differentially expressed genes (DEGs) that were significantly upregulated (Fig. 1A). Functional enrichment analysis of these DEGs highlighted immune-related pathways, particularly macrophage activation (Fig. 1B). Correlation analysis showed that PLA2G7 expression positively correlated with BMI and serum lipids (TC and TG), and negatively correlated with lung function indices (FEV1/FVC and FEV1% predicted) (Fig. 1C–G). Single-cell clustering analysis of lung tissues from high-fat diet (HFD) and control mice identified distinct cell populations, with differential expression of the key gene PLA2G7 in macrophages (F ig. 1H–I). The single-cell RNA sequencing data used in this study are available from NCBI under the accession number Series GSE216695. Immunofluorescence staining further localized PLA2G7 expression to pulmonary macrophages in the lungs of obese COPD patients (Fig. 1J–L).

### Obesity-driven COPD phenotypes are accompanied by PLA2G7 upregulation in mice

**Fig. 2:**
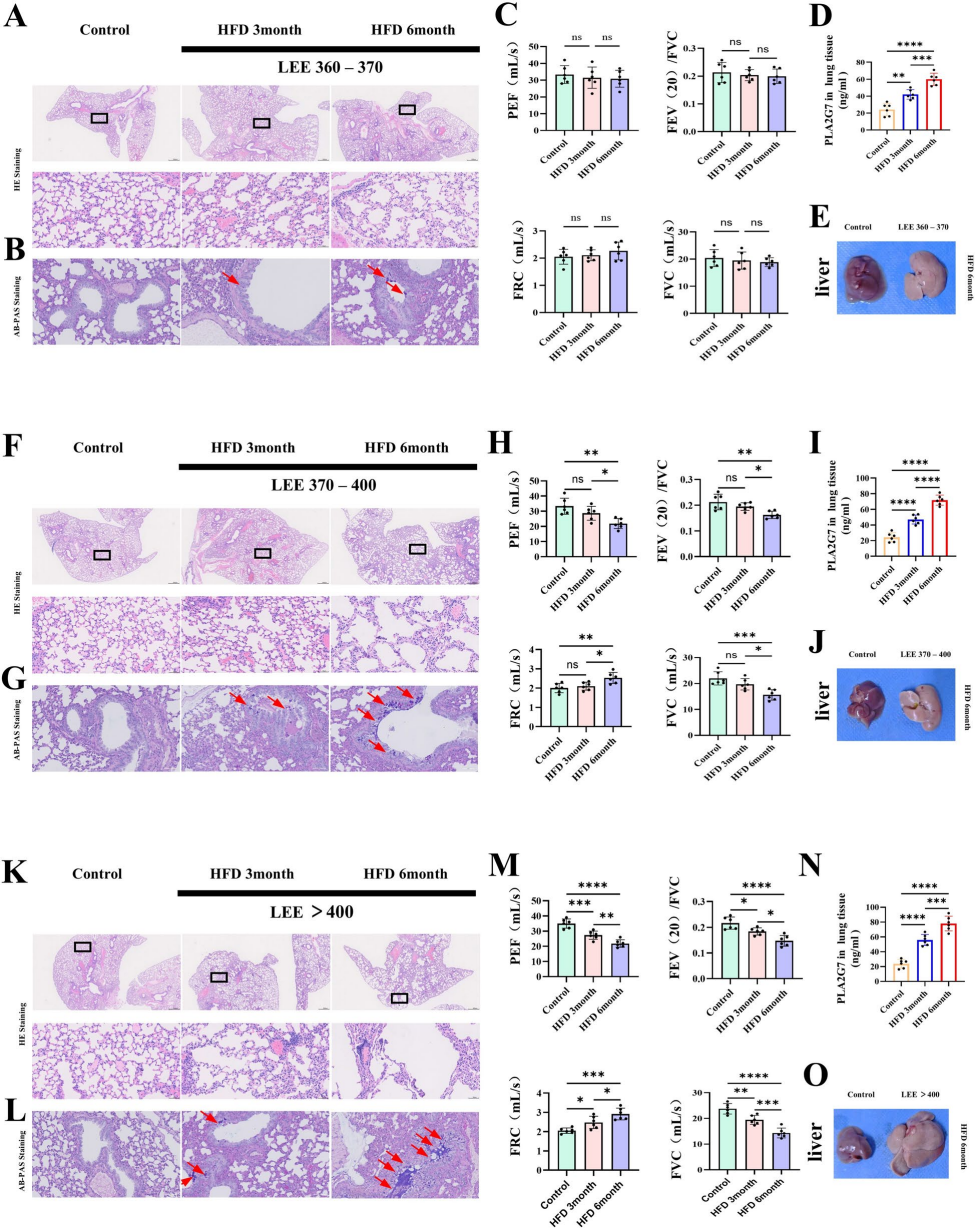
Obesity-associated COPD phenotypes correlate with progressive *PLA2G7* upregulation in mice. **A**-**E** In mildly obese mice (Lee's index 360–370), alveolar integrity and lung function were maintained, and mucus hypersecretion was absent, although *PLA2G7* expression gradually increased with the duration of HFD exposure. **F**-**J** Moderately obese mice (Lee's index 370–400) showed further elevation of *PLA2G7* level; lung architecture remained intact at 3 months, but prolonged HFD feeding (6 months) resulted in alveolar injury, mucus deposition, and reduced lung function. **K**–**O** Severely obese mice (Lee’s index > 400) showed extensive alveolar destruction, severe impairment of pulmonary function, prominent mucus hypersecretion, peak *PLA2G7* expression, and aggravated hepatic steatosis

To assess the impact of obesity on COPD phenotypes, mice were fed a high-fat diet (HFD) for 3 or 6 months and stratified by Lee’s index. Mildly obese mice (Lee’s index 360–370) preserved alveolar structure and lung function, with no overt mucus hypersecretion, although *PLA2G7* expression increased progressively with HFD duration (Fig. 2A-E). Moderately obese mice (Lee’s index 370–400) showed further *PLA2G7* upregulation; while lungs remained intact at 3 months, prolonged HFD (6 months) resulted in evident alveolar damage, mucus accumulation, and functional decline (Fig. 2F-J). In the most obese group (Lee’s index > 400), chronic HFD induced severe alveolar destruction, profound functional impairment, marked mucus hypersecretion, maximal *PLA2G7* expression, and exacerbated hepatic steatosis(Fig. 2K-O). Collectively, these findings establish that obesity drives COPD-like pathology in a body weight–dependent manner, with *PLA2G7* upregulation emerging as a consistent molecular signature.

### PLA2G7 Upregulation disrupts arachidonic acid metabolism and elevates oxidative stress

**Fig. 3:**
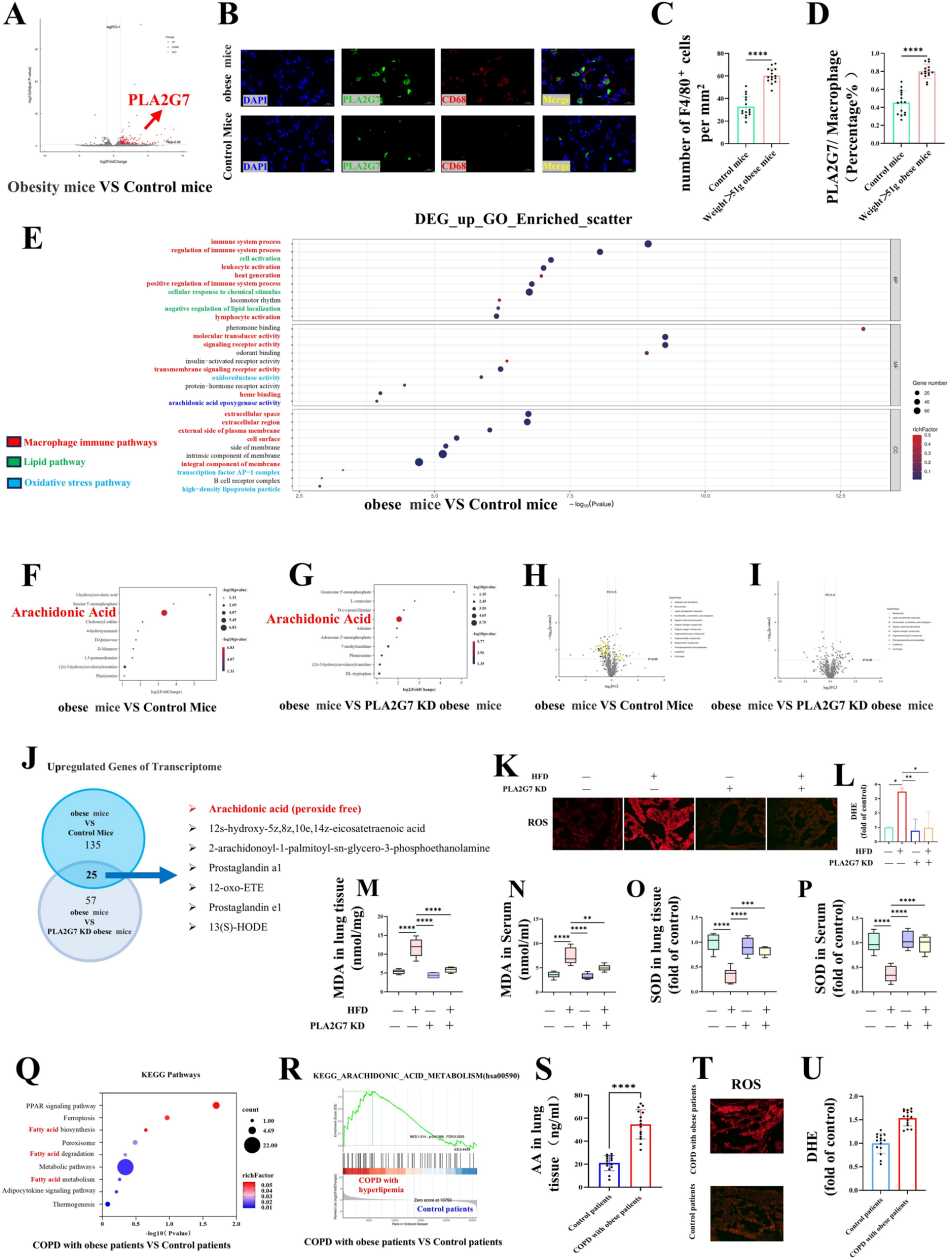
*PLA2G7* upregulation perturbs arachidonic acid metabolism and promotes oxidative stress. **A** RNA-seq analysis of lungs from HFD-fed mice identified PLA2G7 as one of the most significantly upregulated genes. **B**–**D** Immunofluorescence staining showed macrophage infiltration with strong *PLA2G7* colocalization. **E** Functional enrichment of upregulated DEGs indicated pathways related to macrophage activation, fatty acid metabolism, oxidative stress, and AA metabolism. **F**-**J** Metabolomic profiling revealed increased AA and its derivatives in obese lungs, which were significantly reduced after *PLA2G7* knockdown. **K**–**P** HFD-induced ROS production and lipid peroxidation in mouse lungs were decreased by *PLA2G7* silencing. **Q**–**R** KEGG enrichment analysis of upregulated DEGs in human obese COPD lungs highlighted activation of fatty acid and AA metabolism. **S**-**U** Human obese COPD lungs showed elevated AA levels and increased oxidative stress

RNA-seq analysis of obese mouse lungs identified *PLA2G7* as one of the most significantly upregulated genes following HFD exposure (Fig. 3A). Immunofluorescence confirmed extensive macrophage infiltration with strong PLA2G7 expression colocalized in these immune cells (Fig. 3B–D). Enrichment analysis linked the upregulated DEGs to macrophage activation, fatty acid metabolism, and oxidative stress, with arachidonic acid metabolism prominently enriched (Fig. 3E). Metabolomic profiling further demonstrated elevated AA and its derivatives in obese lungs, whereas *PLA2G7* silencing markedly attenuated these increases (Fig. 3F–J). Consistently, HFD feeding induced higher ROS production and lipid peroxidation, both of which were suppressed by *PLA2G7* knockdown (Fig. 3K–P).

Human obese COPD lungs exhibited a similar molecular signature: KEGG enrichment of upregulated DEGs revealed broad activation of fatty acid metabolism with a central role of AA metabolism (Fig. 3Q–R). This was accompanied by increased AA accumulation and heightened oxidative stress (Fig. 3S–U).

Together, these findings establish *PLA2G7* as a conserved regulator of obesity-driven AA metabolic reprogramming and pulmonary oxidative stress, thereby contributing to COPD pathogenesis.

### PLA2G7 mediates obesity-driven pyroptosis in pulmonary tissue

**Fig. 4:**
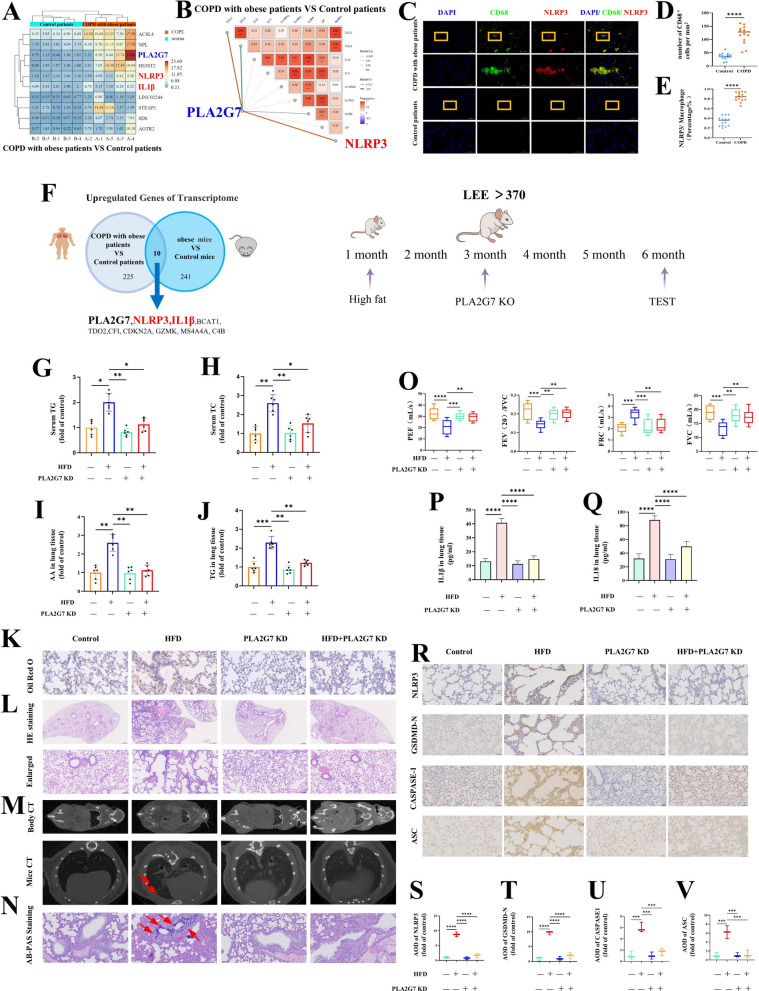
*PLA2G7* mediates obesity-induced pyroptosis in pulmonary tissue. **A** Transcriptomic profiling of human lung tissue revealed upregulation of *PLA2G7, NLRP3*, and *IL1β* in obese COPD patients. **B** Correlation analysis showed positive associations between *PLA2G7* expression and both *NLRP3* and pro-inflammatory cytokines. **C**–**E** Immunofluorescence staining showed colocalization of NLRP3 with macrophages in obese COPD lungs. **F** Cross-species comparison identified ten conserved transcripts consistently upregulated in humans and mice, including PLA2G7, NLRP3, and IL1β. **G**–**H** Circulating lipid levels were reduced in *PLA2G7-*deficient obese mice. **I**–**J** Pulmonary accumulation of AA and triglycerides was decreased in *PLA2G7*-deficient obese mice. **K–M** Histological examination and micro-CT imaging showed preserved alveolar structure without emphysematous changes in PLA2G7-deficient obese mice. **N** Airway mucus secretion was lower in *PLA2G7*-deficient obese mice. **O** Pulmonary function tests indicated improved lung function without *PLA2G7*. **P**–**V** Levels of pyroptosis-related proteins and secretion of pro-inflammatory cytokines were decreased in PLA2G7-deficient obese mice

Transcriptomic profiling of human lung tissue revealed that *PLA2G7*, *NLRP3*, and *IL1β* were significantly upregulated in obese patients with COPD (Fig. 4A). Correlation analysis further demonstrated a strong positive association between *PLA2G7* and *NLRP3* as well as related pro-inflammatory cytokines (Fig. 4B). Immunofluorescence analysis showed marked colocalization of *NLRP3* with macrophages in the lungs of obese COPD patients (Fig. 4C–E). Cross-species analysis identified ten conserved transcripts shared between humans and mice, among which *PLA2G7*, *NLRP3*, and *IL1β* were consistently upregulated (Fig. 4F).

To evaluate the role of *PLA2G7* in obesity-induced lung injury, obese mice (HFD feeding for 3 months, Lee’s index > 370) were randomized to continue HFD feeding or to receive *PLA2G7* deletion followed by 3 months of HFD. Compared with HFD-fed controls, *PLA2G7*-deficient mice exhibited lower circulating lipid levels (Fig. 4G–H), decreased pulmonary accumulation of arachidonic acid and triglycerides (Fig. 4I–J), preserved alveolar architecture without emphysematous changes on histology and micro-CT (Fig. 4K–M), reduced mucus hypersecretion (Fig. 4N), and improved pulmonary function (Fig. 4O). These findings identify *PLA2G7* as a lipid-responsive mediator that drives metabolic stress–induced lipid accumulation and emphysematous remodeling, thereby promoting COPD-like pathology.

Importantly, loss of *PLA2G7* also blunted HFD-induced upregulation of pyroptosis -associated proteins and the release of pro-inflammatory cytokines in the lung (Fig. 4P–V). Together, these results demonstrate that *PLA2G7* promotes NLRP3 inflammasome activation under obese conditions, thereby exacerbating pyroptosis and driving COPD progression.

### PLA2G7 knockdown attenuates AA levels, ROS accumulation, and pyroptosis in OA-stimulated macrophages

**Fig. 5:**
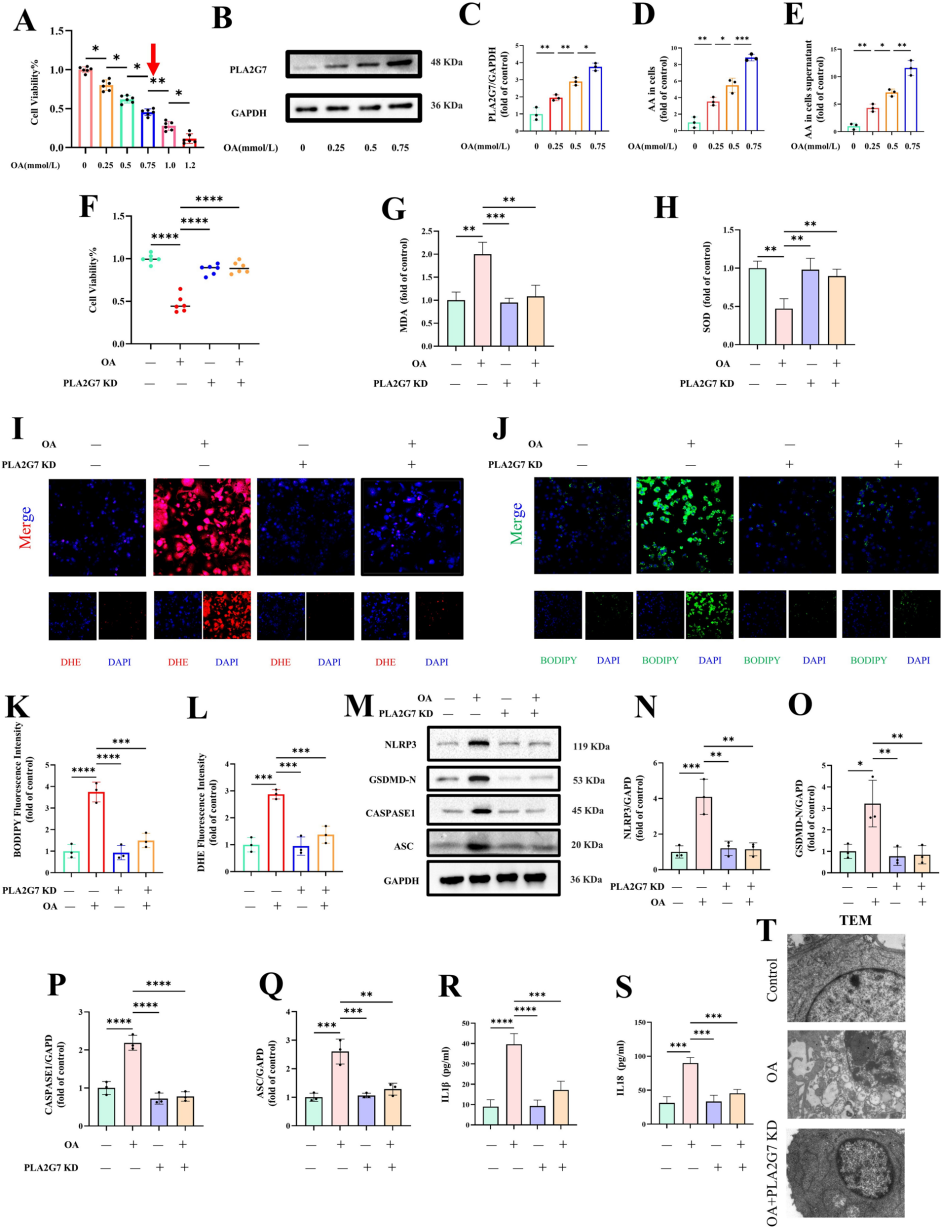
*PLA2G7* knockdown attenuates AA levels, ROS accumulation, and pyroptosis in OA-stimulated macrophages. **A** CCK-8 assay showing dose-dependent reduction in cell viability upon OA exposure. **B**–**C** Western blot analysis demonstrating increased *PLA2G7* expression with OA treatment. **D**–**E** ELISA detection of intracellular and extracellular AA levels, elevated in response to OA. **F** Improved cell viability in *PLA2G7*-silenced macrophages exposed to OA. **G**–**L** Reduced intracellular ROS production and lipid peroxidation following *PLA2G7* knockdown. **M**–**S** OA-induced upregulation of pyroptosis-related proteins and pro-inflammatory cytokines, markedly suppressed in *PLA2G7*-deficient cells. **T** TEM images showing pyroptotic morphology in OA-treated macrophages, absent after *PLA2G7* silencing

To examine the role of *PLA2G7* in lipid-induced macrophage injury, macrophages were exposed to increasing concentrations of OA. OA exposure led to a dose-dependent decrease in cell viability, as shown by the CCK-8 assay (Fig. 5A), along with increased PLA2G7 protein expression (Western blot; Fig. 5B–C). ELISA analysis further indicated a dose-dependent rise in both intracellular and extracellular AA levels (Fig. 5D–E), suggesting that OA-induced lipid overload enhances PLA2G7 expression and promotes AA accumulation. Silencing *PLA2G7* before OA exposure significantly improved cell viability (Fig. 5F). Consistent with in vivo results, *PLA2G7* knockdown decreased OA-induced intracellular ROS production and lipid peroxidation (Fig. 5G–L). Moreover, OA increased pyroptosis-related protein levels and pro-inflammatory cytokine secretion, both of which were substantially reduced in *PLA2G7*-deficient macrophages (Fig. 5M–S). Transmission electron microscopy also showed typical pyroptotic morphology in OA-treated cells, which was absent after *PLA2G7* silencing (Fig. 5T).These results demonstrate that under OA stimulation, *PLA2G7* drives AA and lipid ROS accumulation, thus activating the NLRP3 inflammasome and triggering macrophage pyroptosis.

### Exogenous arachidonic acid restores macrophage pyroptosis suppressed by PLA2G7 silencing

**Fig. 6:**
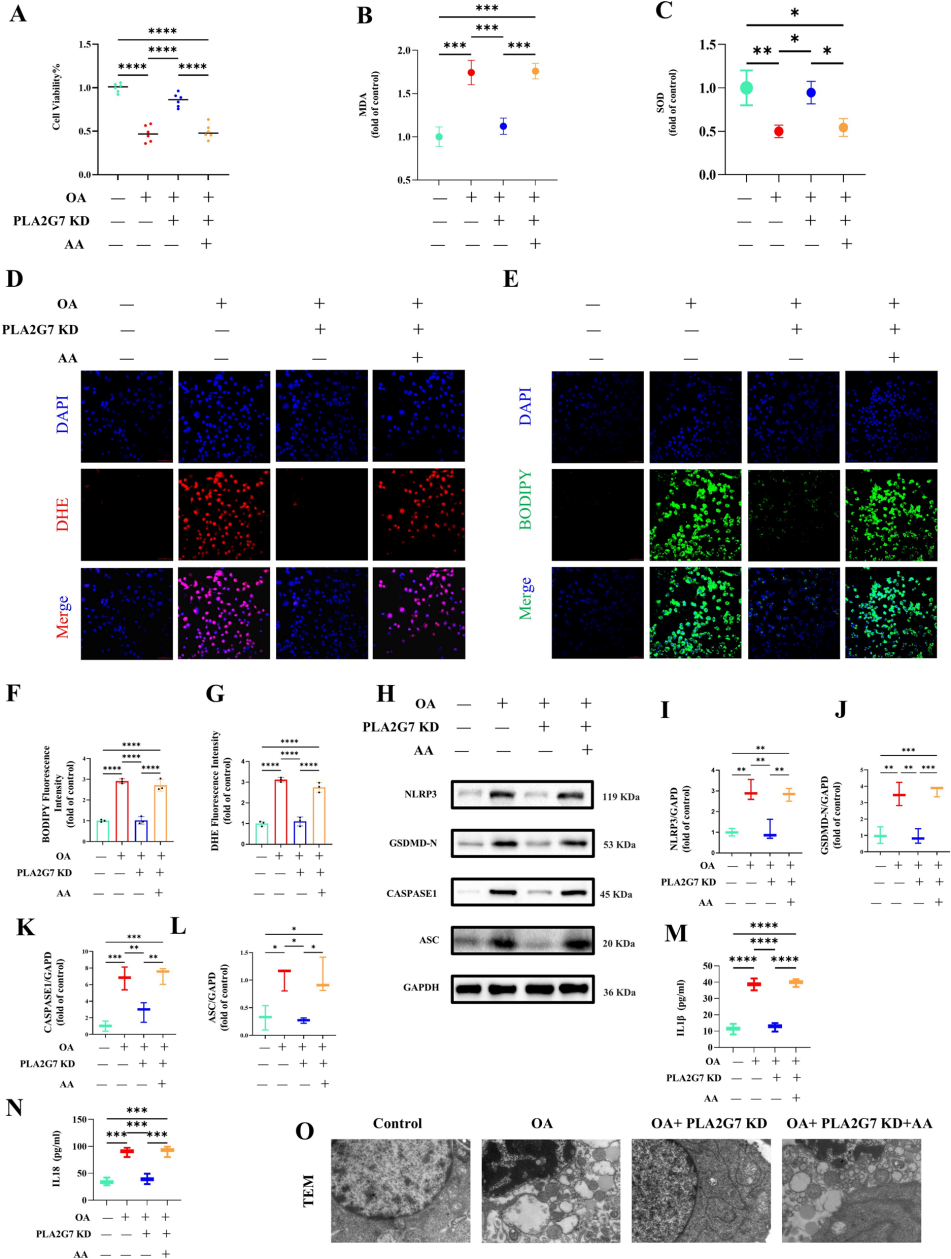
Exogenous arachidonic acid restores macrophage pyroptosis suppressed by *PLA2G7* silencing. (**A**) CCK-8 assays showing that *PLA2G7* silencing alleviated OA-induced cytotoxicity, which was reversed by AA supplementation. (**B**–**G**) Intracellular ROS production and lipid peroxidation reduced by *PLA2G7* deficiency, restored upon AA treatment. (**H**–**N**) Suppression of OA-induced pyroptosis-related proteins and pro-inflammatory cytokine secretion in *PLA2G7*-deficient macrophages, reversed with AA supplementation. (**O**) TEM images confirming pyroptotic morphology in OA-treated cells, absent in *PLA2G7*-silenced cells but reappearing with exogenous AA addition

Macrophages were transfected with *PLA2G7*-specific siRNA for 24 h, followed by treatment with OA for another 24 h. Cells were then incubated in fresh medium supplemented with 30 μM exogenous AA for 24 h. CCK-8 assays showed that knocking down *PLA2G7* significantly reduced OA-induced cytotoxicity, while AA supplementation reversed this protective effect (Fig. 6A). Furthermore, *PLA2G7* deficiency attenuated intracellular ROS generation and lipid peroxidation, effects that were again reversed by AA treatment (Fig. 6B–G). Similarly, OA-induced increases in pyroptosis-associated proteins and secretion of pro-inflammatory cytokines were suppressed in *PLA2G7*-deficient cells but restored with AA addition (Fig. 6H-N). Transmission electron microscopy further confirmed typical pyroptotic morphology in OA-treated cells, which was absent in *PLA2G7*-silenced cells but reappeared following exogenous AA supplementation (Fig. 6O). These observations indicate that under conditions of lipid stress, *PLA2G7* upregulation promotes the accumulation of intracellular AA, which in turn drives excess ROS, and lipid peroxidation, ultimately activating the NLRP3 inflammasome and triggering macrophage pyroptosis.

### Arachidonic acid slows NLRP3 mRNA degradation

**Fig. 7:**
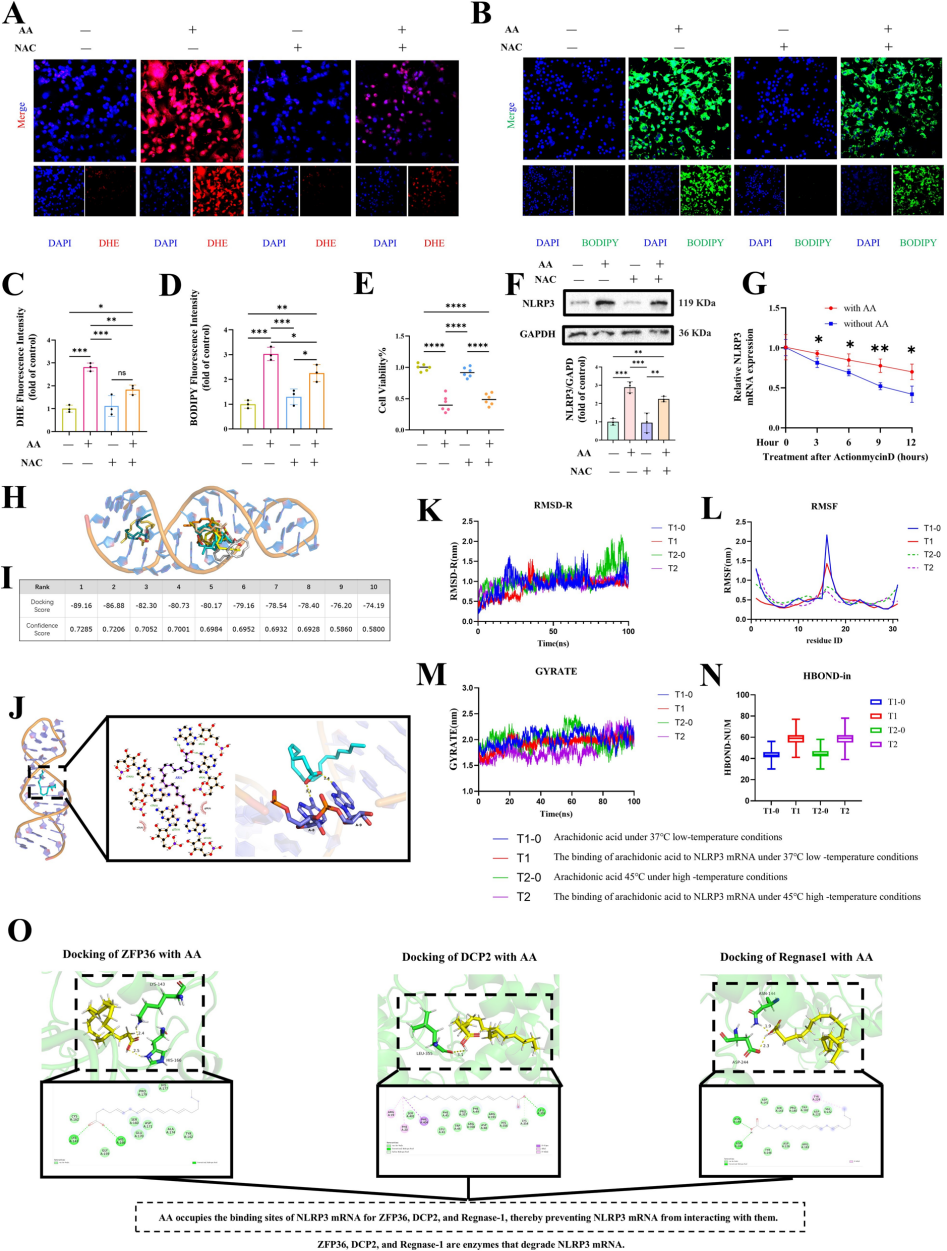
Arachidonic acid stabilizes NLRP3 mRNA by reducing degradation **A**-**D** Treatment with AA (30 μM, 24 h) increased lipid ROS levels in alveolar macrophages, an effect partially reversed by NAC (5 mM). **E** Partial cell death was observed despite the inhibition of ROS. **F **NLRP3 protein expression showed a tendency to decrease after NAC exposure. **G **Actinomycin D assays demonstrated a delay in the degradation of NLRP3 mRNA in the presence of AA. **H**-**J **Molecular docking analysis revealed a strong interaction between AA and NLRP3 mRNA. **K**-**N **Molecular dynamics simulations indicated that AA reduced RMSD fluctuations, decreased nucleotide mobility (RMSF), maintained structural compactness (Rg), and enhanced intramolecular hydrogen bonding. **O **AA occupied the binding regions of ZFP36, DCP2, and Regnase-1 on NLRP3 mRNA, thus preventing their binding

Exposure of alveolar macrophages to AA (30 μM, 24 h) markedly increased lipid ROS levels, whereas co-treatment with the ROS inhibitor NAC (5 mM) attenuated this effect (Fig. 7A–D). Despite the reduction in lipid ROS, CCK-8 assays revealed that partial cell death still occurred (Fig. 7E), and NLRP3 protein expression showed a decreasing trend upon NAC treatment (Fig. 7F). These findings suggest that AA-induced *NLRP3* upregulation is not solely mediated by ROS, implicating additional mechanisms.

To investigate this, we performed actinomycin D(ActD) chase assays to assess *NLRP3* mRNA stability. Alveolar macrophages were cultured in complete medium and treated with ActD to block transcription, followed by incubation with or without exogenous AA. *NLRP3* mRNA levels were measured at 0, 3, 6, 9, and 12 h after ActD treatment. The results showed that AA treatment significantly enhanced the stability of *NLRP3* mRNA, leading to a slower degradation rate (Fig. 7G). To further explore the underlying mechanism, molecular docking analyses were conducted, which revealed strong binding affinity between AA and *NLRP3* mRNA (Fig. 7H–J).

Molecular dynamics simulations confirmed that AA binding contributed to mRNA structural stabilization. Specifically, RMSD analyses demonstrated reduced conformational fluctuations in AA-bound *NLRP3* mRNA at the same temperature (Fig. 7K). RMSF profiles indicated decreased flexibility of individual nucleotides upon AA binding (Fig. 7L). Gyration radius analyses further revealed more stable compaction–expansion behavior of the mRNA structure in the presence of AA (Fig. 7M). Consistently, intramolecular hydrogen bond analysis showed that AA promoted an increased number of internal hydrogen bonds within *NLRP3* mRNA, further supporting its stabilizing effect (Fig. 7N).

Moreover, *NLRP3* mRNA degradation is known to be regulated by ZFP36, DCP2, and Regnase-1. Strikingly, docking results demonstrated that AA occupied the binding sites of these enzymes on *NLRP3* mRNA, thereby preventing their interaction (Fig. 7O). Collectively, these findings indicate that AA stabilizes *NLRP3* mRNA by both enhancing its structural integrity and blocking the access of degradation enzymes, ultimately slowing down *NLRP3* mRNA decay.

### NLRP3 overexpression reverses the protective effects of PLA2G7 knockdown under lipid stress in vitro

**Fig. 8:**
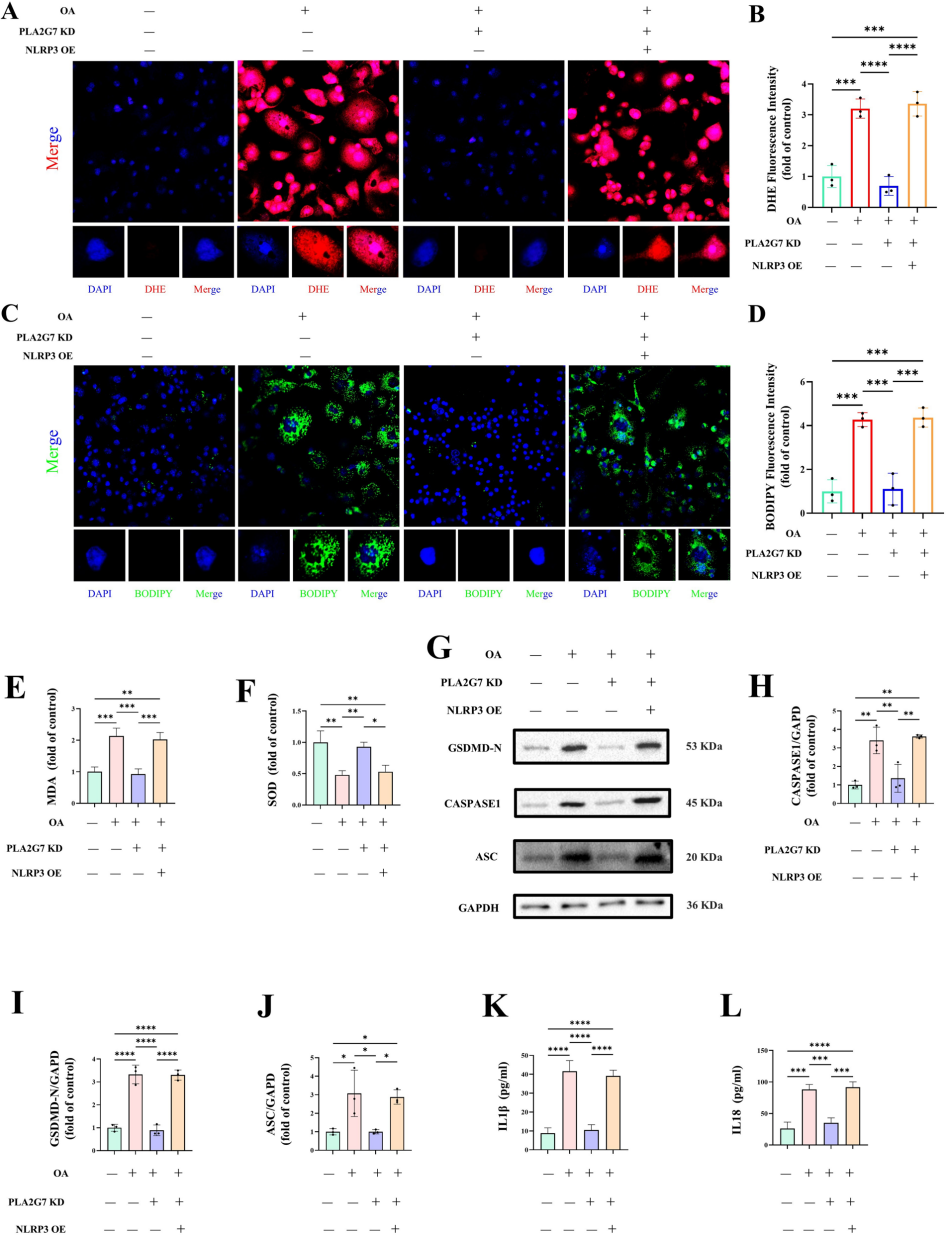
*NLRP3* overexpression reverses the protective effects of *PLA2G7* knockdown under lipid stress in vitro. **A**–**F** Intracellular ROS (DHE staining) and lipid peroxidation (BODIPY staining, MDA, and SOD assays) were reduced by *PLA2G7* knockdown in OA-treated macrophages, and these effects were reversed mainly by *NLRP3* overexpression. **G**–**L** *PLA2G7* deficiency–mediated suppression of pyroptosis-related protein expression (Western blot) and pro-inflammatory cytokine release (ELISA) was restored upon NLRP3 overexpression

To investigate whether *NLRP3* mediates *PLA2G7*-dependent pyroptosis under lipid stress, alveolar macrophages were transfected with *PLA2G7* siRNA prior to OA exposure, with or without concomitant *NLRP3* overexpression. *PLA2G7* silencing significantly attenuated OA-induced ROS production and lipid peroxidation, whereas *NLRP3* overexpression largely restored these effects (Fig. 8A–F).

Similarly, *PLA2G7* knockdown suppressed the induction of pyroptosis-related proteins and the secretion of proinflammatory cytokines following OA exposure. Notably, these inhibitory effects were reversed by *NLRP3* overexpression (Fig. 8G–L).

Collectively, these results indicate that *NLRP3* acts downstream of *PLA2G7* and is a key effector that promotes macrophage pyroptosis in response to lipid stress.

### PLA2G7 suppression reduces lung injury in obese mice

**Fig. 9:**
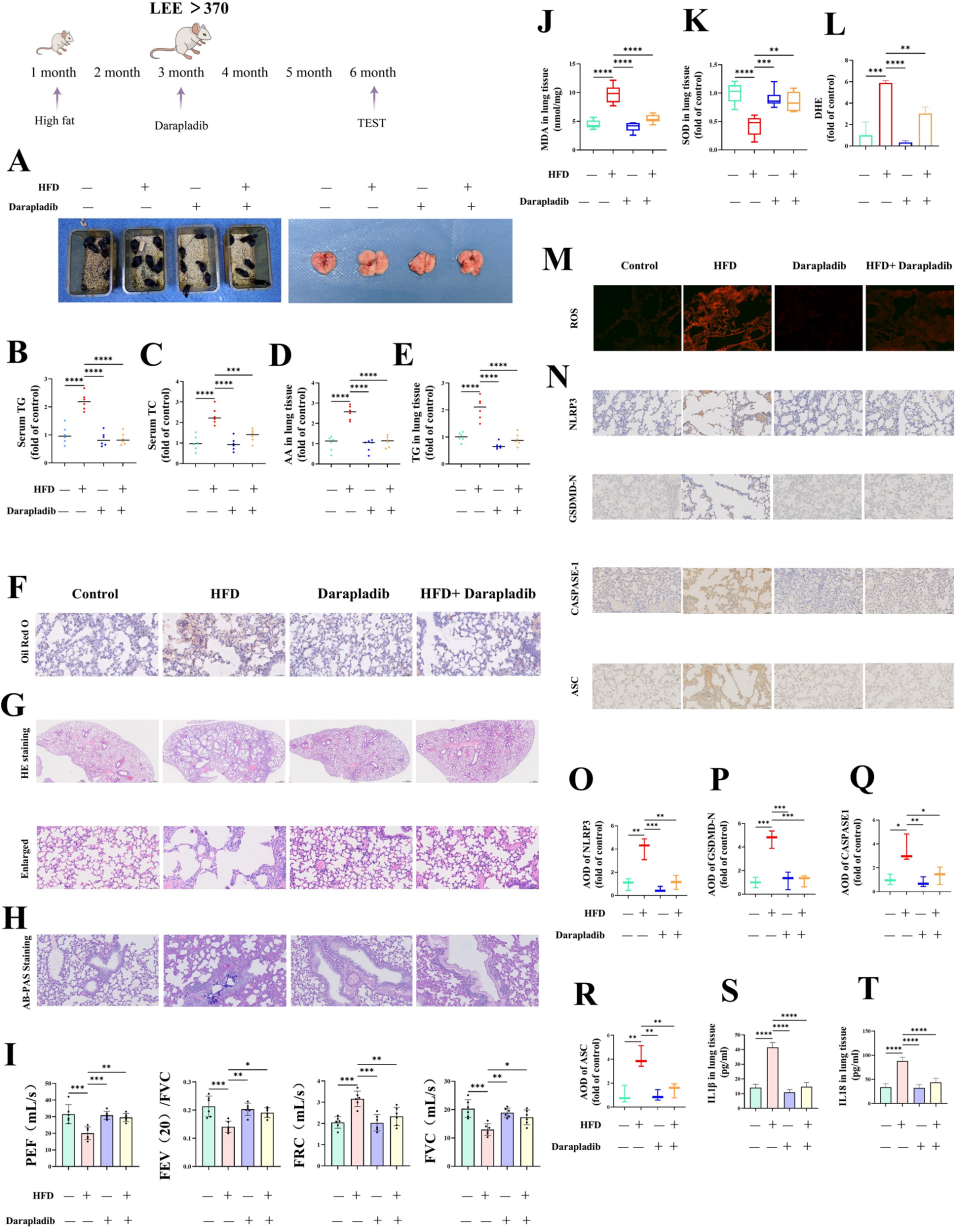
*PLA2G7* suppression reduces lung injury in obese mice. **A**–**C** Darapladib treatment reduced serum TG and TC levels compared with HFD-fed controls. **D** Pulmonary AA content was decreased in Darapladib-treated mice. **E**–**F** Pulmonary TG accumulation was reduced, confirmed by Oil Red O staining and biochemical quantification. **G** H&E staining showed no emphysematous changes in Darapladib-treated lungs. **H** AB-PAS staining indicated reduced airway mucin secretion in Darapladib-treated lungs. **I** Lung function was improved after *PLA2G7* inhibition. **J**–**M** Darapladib attenuated HFD-induced ROS accumulation (DHE staining), elevated MDA levels, and decreased SOD activity. **N**–**R** Pulmonary expression of pyroptosis-related proteins was reduced in Darapladib-treated mice (immunohistochemistry).(S–T) *PLA2G7* knockdown reduced IL-1β and IL-18 levels (ELISA). Together, these data indicate that PLA2G7 overactivation drives obesity-associated pulmonary injury, and pharmacological inhibition effectively alleviates structural, functional, and inflammatory lung damage

To evaluate the direct role of *PLA2G7* in obesity-related lung damage, obese mice (HFD feeding for 3 months, Lee’s index > 370) were fed an HFD with or without daily oral doses of the PLA2G7 inhibitor Darapladib (50 mg/kg). Treatment with Darapladib significantly reduced HFD-induced lung injury, shown by lower levels of circulating triglycerides and total cholesterol (Fig. 9A-C) and a significant decline in pulmonary AA content (Fig. 9D). Pulmonary triglyceride accumulation was also decreased, as confirmed by Oil Red O staining and biochemical analysis (Fig. 9E-F). H&E staining revealed that alveolar structure was preserved without signs of emphysema (Fig. 9G). AB-PAS staining revealed that pharmacological intervention significantly reduced airway mucin secretion (Fig. 9H). Lung function tests showed substantial improvement compared to untreated HFD-fed mice (Fig. 9I). These findings suggest that PLA2G7 activity is a key factor driving airway mucin secretion and emphysematous remodeling in the lungs of obese mice.

DHE fluorescence showed excessive ROS production, along with increased MDA and decreased SOD activity; all these changes were significantly reduced by PLA2G7 inhibition (Fig. 9J-M). Furthermore, obesity was associated with higher expression of pyroptosis-related proteins (Fig. 9N-R) and increased secretion of IL-1β and IL-18 (Fig. 9S-T), both of which were decreased by Darapladib treatment.

These findings demonstrate that PLA2G7 overactivation in obese lungs leads to COPD-like structural and functional impairment. Pharmacologically blocking PLA2G7 effectively interrupts this pathogenic pathway, offering therapeutic benefit against obesity-related pulmonary injury.

### PLA2G7 suppression reduces pyroptosis in OA-stimulated macrophages

**Fig. 10:**
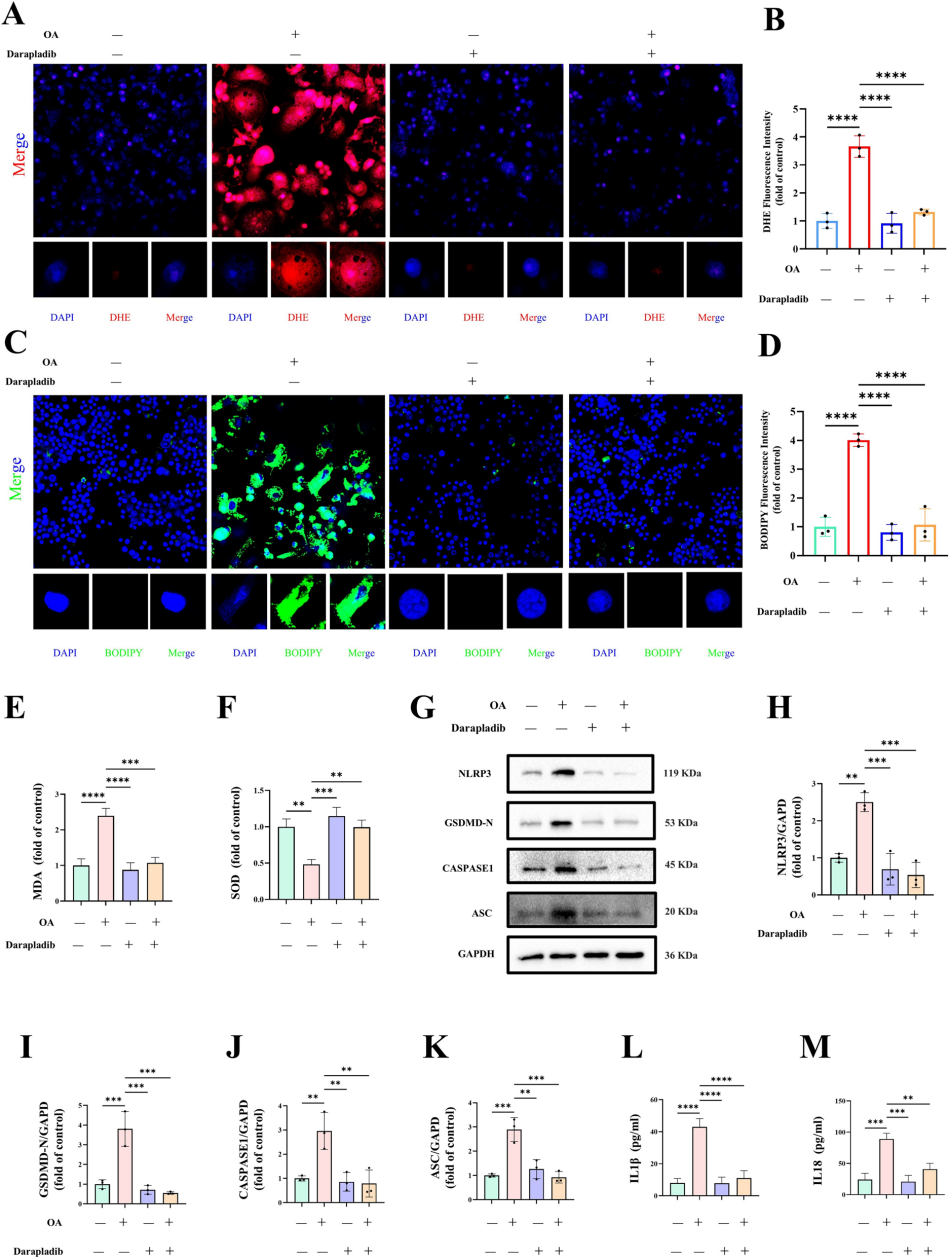
*PLA2G7* suppression reduces pyroptosis in OA-stimulated macrophages. **A**–**F** Intracellular ROS levels (assessed by DHE staining) and lipid peroxidation (evaluated by BODIPY staining, MDA, and SOD assays) were significantly reduced by Darapladib in OA-treated macrophages. **G**–**M** Darapladib also suppressed pyroptosis-related protein expression (Western blot) and reduced the release of pro-inflammatory cytokines (ELISA)

To further investigate the protective role of PLA2G7 inhibition in macrophages under lipid stress, cells were pretreated with the selective PLA2G7 inhibitor Darapladib prior to OA exposure. Darapladib markedly attenuated OA-induced ROS production and lipid peroxidation (Fig. 10A–F). Consistently, Darapladib treatment suppressed the induction of pyroptosis-associated proteins and significantly reduced the secretion of proinflammatory cytokines following OA stimulation (Fig. 10G–M). These findings indicate that pharmacological inhibition of PLA2G7 effectively restrains lipid stress–induced macrophage pyroptosis.

## Discussion

The "obesity paradox" presents a complex clinical challenge in the management of COPD [26, 27]. While obesity is strongly associated with comorbidities, a higher body mass index (BMI ≥ 30 kg/m^2^) has often been associated with lower death rates [28]. However, this protective effect is not consistent [29]. Increasing evidence suggests a U-shaped relationship, where the survival benefit decreases or vanishes once the BMI exceeds a certain threshold (≥ 35 kg/m^2^) [30, 31]. At this level, the systemic metabolic burden of obesity appears to outweigh its beneficial effects, as reflected by greater susceptibility to acute exacerbations and higher rates of hospitalizations in severely obese COPD patients [32, 33]. These nonlinear relationships underscore the importance of stratified clinical strategies and suggest that individuals with a BMI of 35 kg/m^2^ or higher may require more precise and targeted treatments [34–36].

Despite extensive clinical observations, the causal pathways through which obesity contributes to COPD pathogenesis remain poorly understood [37]. It also remains uncertain whether severe obesity alone is enough to directly trigger the disease [38]. Experimental results from this study provide evidence that obesity itself can provoke COPD-like changes in the lung. In a diet-induced obesity model, increasing body weight was associated with alveolar destruction, mucus hypersecretion and a decrease in lung function. When the mouse body weight exceeded 51 g (Lee’s index > 400), approximately corresponding to a human BMI of ≥ 35 kg/m^2^, distinct COPD-like phenotypes became apparent. This model offers a novel platform for investigating the pathobiology of obesity-associated COPD.

A combination of transcriptomic analyses from the lungs of obese COPD patients and obese mouse models, along with macrophage-focused in vitro studies, identified *PLA2G7* as a key macrophage-derived mediator of obesity-driven COPD. Obese mice showed body weight–dependent *PLA2G7* upregulation. Lipid stress–induced *PLA2G7* expression in macrophages increased AA levels, which enhanced membrane susceptibility to peroxidation and increased lipid ROS production. Simultaneously, AA enhanced the stability of *NLRP3* mRNA, leading to a slower degradation process. This cascade activated the NLRP3 inflammasome and caused pyroptotic cell death, leading to aggravated pulmonary inflammation and accelerated COPD progression. These results outline a comprehensive mechanistic framework that connects lipid metabolism, innate immune signaling, and regulated cell death in obesity-associated lung disease.

From a translational perspective, in vivo evidence highlights *PLA2G7* as a promising therapeutic target. Both pharmacological blockade and genetic deficiency of *PLA2G7* reduced obesity-related COPD-like pathology in mice, highlighting its potential as a treatment option. Targeting macrophage-mediated inflammatory pathways at the intersection of metabolic and immune pathways may represent a novel strategy to attenuate disease severity in metabolically driven COPD phenotypes.

This study also addresses a significant gap in current knowledge. Previous studies have examined mainly systemic metabolic disturbances or localized inflammatory responses separately, without clarifying the role of specific metabolic enzymes in immune cells that combine these signals to promote pulmonary injury [39, 40]. By positioning *PLA2G7* at the intersection of metabolic stress and inflammatory cell death, the findings establish a unified mechanistic framework that connects previously disparate observations in obesity-related immunometabolic dysregulation.

This study provides valuable insights into the mechanistic link between obesity and COPD, focusing on PLA2G7 as a mediator in obesity-related lung injury. However, several limitations should be considered when interpreting the findings, particularly regarding causality, cell-type specificity, and therapeutic relevance. The lack of a pair-fed control group limits our ability to establish causality between obesity and the observed pulmonary damage. High-fat diet (HFD) feeding itself can contribute to lung inflammation, making it difficult to separate the effects of obesity from diet-induced changes. Future studies with pair-fed HFD cohorts and alternative obesity models are needed to confirm the causal role of obesity in COPD-like pathology. Although macrophages are identified as key mediators, the specificity of PLA2G7 expression to macrophages remains uncertain. The absence of conditional Pla2g7 knockouts and single-cell analyses prevents definitive attribution of the phenotype to macrophages alone. Future research incorporating cell-specific knockouts and comprehensive single-cell analysis is required to explore the roles of other cell types, including epithelial, endothelial, and mesenchymal cells.The mechanism by which arachidonic acid (AA) stabilizes NLRP3 mRNA is based on in silico predictions, with no direct biochemical evidence to confirm this interaction. Future studies should include biochemical assays to validate AA–NLRP3 binding and investigate its impact on RNA decay machinery. The use of Darapladib as a PLA2G7 inhibitor raises the possibility that its effects on systemic lipid levels and inflammation may indirectly alleviate lung injury. The lack of localized delivery or cell-targeted approaches limits our ability to attribute the benefits to lung-specific PLA2G7 inhibition. Targeted drug delivery systems should be employed in future studies to better isolate the effects of PLA2G7 inhibition within the lung microenvironment. The clinical component of this study is limited by a small sample size, reducing statistical power and generalizability. Furthermore, key confounders, including age, sex, occupational exposures, metabolic comorbidities, and secondary smoking, were not controlled for. Larger clinical studies with more comprehensive data on these factors are needed to enhance the relevance of these findings to broader populations.

## Conclusion

**Fig. 11:**
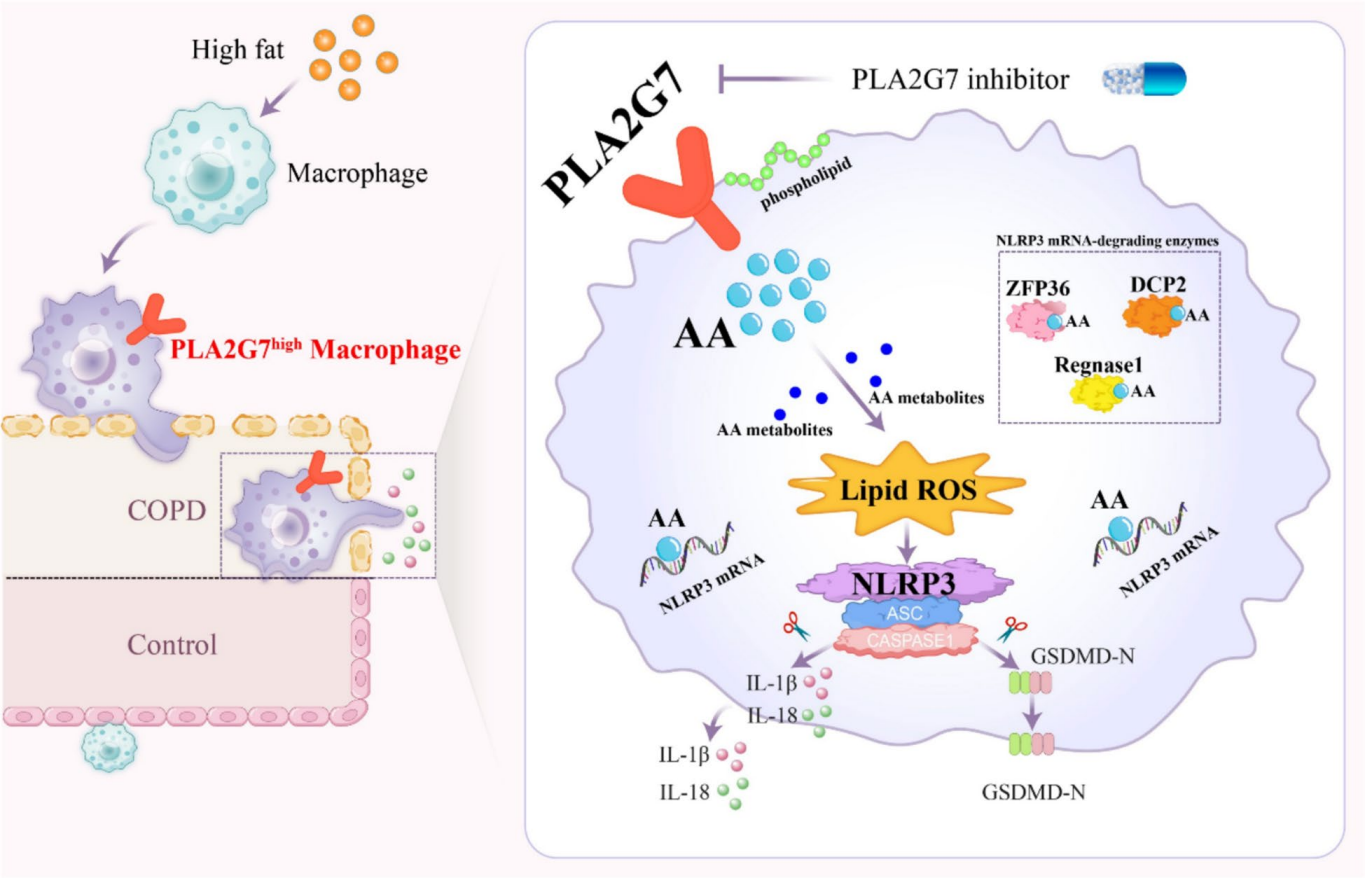
PLA2G7-Orchestrated Metabolic–Immune Reprogramming in Obesity-Associated COPD. Obesity-driven upregulation of PLA2G7 increases arachidonic acid levels, thereby promoting lipid ROS accumulation and stabilizing NLRP3 mRNA by preventing its decay. These events facilitate NLRP3 inflammasome activation and induce pyroptosis, providing mechanistic insight into metabolic–immune interactions in COPD and underscoring PLA2G7 as a promising therapeutic target in obesity-associated COPD

Collectively, these findings delineate a novel pathogenic axis in which obesity-driven *PLA2G7* upregulation acts as an upstream mediator to increase AA levels. The elevated AA facilitates lipid ROS accumulation and stabilizes *NLRP3* mRNA, which together trigger NLRP3 inflammasome activation and ultimately induce pyroptosis. This mechanistic insight improves the understanding of metabolic–immune interactions in COPD development and identifies *PLA2G7* as a promising therapeutic target for the obesity-associated COPD phenotype. (Fig. 11).

## Data Availability

The authors declare that all data supporting the findings of this study are available from the corresponding author on reasonable request.
